# Dairy Intake and Risk of Cognitive Decline and Dementia: A Systematic Review and Dose-Response Meta-Analysis of Prospective Studies

**DOI:** 10.1016/j.advnut.2023.100160

**Published:** 2023-12-01

**Authors:** Fanny Villoz, Tommaso Filippini, Natalia Ortega, Doris Kopp-Heim, Trudy Voortman, Manuel R. Blum, Cinzia Del Giovane, Marco Vinceti, Nicolas Rodondi, Patricia O. Chocano-Bedoya

**Affiliations:** 1 Institute of Primary Health Care (BIHAM), University of Bern, Bern, Switzerland; 2 Department of General Internal Medicine, Inselspital, Bern University Hospital, University of Bern, Bern, Switzerland; 3 Section of Public Health, Department of Biomedical, Metabolic and Neural Sciences, University of Modena and Reggio Emilia, Modena, Italy; 4 School of Public Health, University of California Berkeley, Berkeley, CA, United States; 5 Population Health Laboratory, University of Fribourg, Fribourg, Switzerland; 6 Public Health and Primary Care Library, University Library of Bern, University of Bern, Bern, Switzerland; 7 Department of Epidemiology, Erasmus MC University Medical Center, Rotterdam, the Netherlands; 8 Department of Epidemiology, Boston University School of Public Health, Boston, MA, United States; 9 Department of Medical and Surgical Sciences for Children and Adults, University-Hospital of Modena and Reggio Emilia, Modena, Italy

**Keywords:** cognitive decline, cohort studies, dairy products, dementia, dose-response meta-analysis

## Abstract

Dairy intake may influence cognition through several molecular pathways. However, epidemiologic studies yield inconsistent results, and no dose-response meta-analysis has been conducted yet.

Therefore, we performed a systematic review with a dose-response meta-analysis about the association between dairy intake and cognitive decline or incidence of dementia.

We investigated prospective studies with a follow-up ≥6 mo on cognitive decline or dementia incidence in adults without known chronic conditions through a systematic search of Embase, Medline, Cochrane Library, Web of Science, and Google Scholar from inception to 11 July 2023. We evaluated the dose-response association using a random-effects model.

We identified 15 eligible cohort studies with >300,000 participants and a median follow-up of 11.4 y. We observed a negative nonlinear association between cognitive decline/dementia incidence and dairy intake as assessed through the quantity of consumption, with the nadir at ∼150 g/d (risk ratio: 0.88; 95% confidence interval: 0.78, 0.99). Conversely, we found an almost linear negative association when we considered the frequency of consumption (risk ratio for linear trend: 0.84; 95% confidence interval: 0.77, 0.92 for 1 time/d increase of dairy products). Stratified analysis by dairy products showed different shapes of the association with linear inverse relationship for milk intake, whereas possibly nonlinear for cheese. The inverse association was limited to Asian populations characterized by generally lower intake of dairy products, compared with the null association reported by European studies.

In conclusion, our study suggests a nonlinear inverse association between dairy intake and cognitive decline or dementia, also depending on dairy types and population characteristics, although the heterogeneity was still high in overall and several subgroup analyses. Additional studies should be performed on this topic, including a wider range of intake and types of dairy products, to confirm a potential preventing role of dairy intake on cognitive decline and identify ideal intake doses.

This review was registered at PROSPERO as CRD42020192395.

**Statements of significance**

This systematic review and meta-analysis identified 15 prospective observational studies evaluating the role of dairy on cognitive function. Our results suggest that dairy might be associated with a lower risk of cognitive decline or dementia but that the relation seems nonlinear with differences by sex, age, region of origin, level of intake, and type of dairy products.

## Introduction

Cognitive decline ranges from the minimal decline that is associated with normal aging to dementia. In between these 2 extremities, Mild Cognitive Impairment corresponds to an intermediate stage [1]. With an overall prevalence of Mild Cognitive Impairment worldwide assessed at 15.6 % in 2022 and an estimated 57.4 million cases of dementia worldwide in 2019 [2], cognitive decline represents a major health issue. Moreover, this burden will be of even greater concern in the future, with a projection of 152.8 million cases of dementia in 2050 [3]. Although no effective treatment is available to counteract dementia progression [4], ≤40% of dementia could be prevented or delayed if addressing modifiable risk factors [5].

Growing evidence from in vitro or in animal models and from individual epidemiologic studies in healthy adults highlights cues of association between nutrition and cognitive function through several mechanisms, including inflammation, oxidative stress, and control of other risk factors [6]. Dairy products may have anti-inflammatory and neuroprotective properties [[7], [8], [9]]. In addition, dairy products might lower the risk of cardiovascular and metabolic disease [10,11], which are known risk factors for cognitive impairment and dementia [12]. Nevertheless, on a meta-analytical level, the association between dairy intake and cognitive function has not been robustly illustrated yet. Previous systematic reviews and meta-analyses have led to conflicting trends [13,14]. On the one hand, the meta-analysis by Wu et al., (2016) [14], including 3 cross-sectional and 4 cohort studies, found that high milk consumption was associated with decreased risk of cognitive disorders [odds ratio (OR): 0.72; 95% confidence interval (CI): 0.56, 0.93]. However, this result was treated with caution in the perspective of many limitations of the study, which were principally the large heterogeneity (I^2^: 64%) because of the type of outcome and characteristics of participants. As a matter of fact, the authors reported a stronger negative association with no heterogeneity (I^2^: 0%) in subjects with Alzheimer’s disease compared to cognitive impairment/decline and overall dementia and in Asian and African populations compared to Caucasian. On the contrary, the more recent systematic review and meta-analysis by Lee et al., (2018) [13] identified 1 randomized controlled trial (RCT) and 7 observational cohort studies. Because of limited reported data, the meta-analysis was conducted only among 3 observational cohort studies. Although the authors reported no association between dairy intake and cognitive decline, their results were in the opposite direction to those of Wu et al. [14], with a higher risk of cognitive decline with higher dairy intake (relative risk: 1.21; 95% CI: 0.81, 1.82, for the highest compared with the lowest intake, I^2^: 64%).

Because additional prospective studies on dairy and cognition have been recently published [[15], [16], [17], [18], [19]], and no dose-response meta-analysis is available, we decided to carry out a new meta-analysis. We also decided to take into account all dairy foods as 1 food group and, whenever possible, subgroups of dairy products, dose-response relationship, geographic differences, and length of follow-up, which could have led to high heterogeneity in previous meta-analyses.

The objective of this systematic review and meta-analysis was to summarize the literature on the association between dairy and cognitive decline or incident dementia and to explore the shape of the association using, whenever possible, dose-response nonlinear modeling.

## Methods

The protocol was registered with the International PROSPERO with the registration number CRD42020192395 and adhered to the PRISMA [20].

### Literature search

We conducted a comprehensive literature search in cooperation with an experienced medical information specialist in Embase.com (Elsevier), Medline (Ovid), Cochrane Central Register of Controlled Trials (Wiley), Cochrane Database of Systematic Reviews (Wiley), Web of Science Core Collection (Clarivate) and Google Scholar, from inception up to 11 July 2023 (last date searched) to identify all prospective observational studies and RCTs that reported data on usual dairy intake at baseline, with prospective follow-up data on cognitive decline or incidence dementia among adults. The search strategy combined terms related to dairy intake (among others, dairy products, milk, yogurt, butter, cheese, cream, whey, casein, and lactalbumin) and cognitive decline (dementia, memory disorder, cognitive defect, Alzheimer’s, and neuro-degenerative disease). No date limits were applied. The full search strategies in all databases are provided in Supplementary Material 1. In addition, we reviewed reference lists of included studies to retrieve additional relevant articles. We removed duplicate records using Deduklick (Risklick), a fully automated deduplication algorithm [21]. The results of the searches were uploaded into Rayyan (https://www.rayyan.ai) [22] for title/abstract screening and full-text evaluation.

### Study selection and data extraction

Two reviewers (FV and TF) independently screened the titles and abstracts of the retrieved studies to exclude articles that did not meet the eligibility criteria. Then, they retrieved full texts of the potentially eligible studies and again assessed their eligibility independently. We included studies only in English and in peer-reviewed journals. We excluded studies that recruited only subjects with chronic conditions (e.g., diabetes, hypertension, metabolic syndrome, dyslipidemia, etc.), cross-sectional studies, and studies with a follow-up of <6 mo. For RCTs, we additionally required that studies have a nondairy or low-dairy control group (i.e., not only comparing different dairy products). We also excluded studies that used nonbovine or human milk interventions. We recorded reasons for exclusion in the full-text screening (Supplementary Material 2). Any disagreement between the authors regarding the eligibility of a study was resolved through discussion with a third reviewer (POC-B). We illustrated the selection process in a PRISMA flow diagram.

Two reviewers (FV and TF) independently extracted multiple fields based on the following categories: general study information (authors, journal, year of publication, and title), study design (country of origin, setting, sample size, and follow-up time), participant characteristics [age, sex, body weight, and BMI (in kg/m^2^)], exposure (dietary assessment and type of dairy), outcome assessment method (cognitive decline or incident dementia), outcome data (effect estimates with measures of variation and covariates). When a study reported stratified analysis only divided by characteristics of the study population (e.g., apolipoprotein E status) or type of outcome (e.g., Alzheimer’s disease and non-Alzheimer’s disease diagnosis), we combined their results using a fixed-effects model and then included them into the analysis comparing the highest compared with the lowest exposure (e.g., forest plots). Conversely, when including study results in the dose-response analysis, we had to consider them as strata-specific study results. From observational studies, we extracted the outcome data from the most adjusted multivariable models. We extracted relative risk or hazard ratio along with 95% CIs for dichotomous outcomes and mean differences and standard deviation/standard error for continuous outcomes. Finally, we asked the authors of 4 studies [[23], [24], [25], [26]] to give us further information on the median dose or ranges in each category or to clarify the definition of serving size. However, we did not receive additional information.

### Data synthesis and analysis

We performed pairwise meta-analyses for all exposures and outcomes using a restricted maximum likelihood random-effects model [27]. We planned to analyze observational studies separately from RCTs. For dichotomous outcomes (cognitive decline or dementia), we computed the summary risk ratio (RR). Results are presented for the combined outcome (i.e., cognitive decline or dementia incidence), and we performed stratified analysis whenever possible (see below subgroup analyses). We have focused our description and interpretation of the results on assessing the size of point estimates and their statistical precision (CIs) measures without *P* value fixed cutpoints [[28], [29], [30]].

We assessed the potential nonlinear relationship through the estimation of a dose-response relationship between dairy intake (measured as the amount in grams/day or frequency in times/day) and cognition. For each exposure category, we assigned the mean or median intake along with the RR and the CI, the number of cases, and of person-years. When means or the median were not available, we used the midpoint of each intake category. For open-ended categories, we used a value 20% lower or higher than the boundary values as performed in other fields [[31], [32], [33]]. For 1 study [15] reporting mean dairy intake in g/1000 kcal/d for each category, we used the mean kcal of the same category to calculate the value in g/d. We used a restricted cubic spline function with 3 knots at fixed cutpoints (10th, 50th, and 90th percentiles) using a restricted maximum likelihood random-effects model [34], assessing the presence of a linear trend [35]. We also presented the results as RR and relative 95% CIs comparing the highest compared with the lowest exposure category in forest plots.

### Subgroup and sensitivity analyses

Whenever possible, we conducted subgroup analysis by type of dairy product, mean age (<65 compared with ≥65 y), sex, region of origin (Asia, Europe, and Oceania), length of follow-up (<10 compared with ≥10 y), and excluding studies at high risk of bias to reveal potential sources of heterogeneity. In addition, we performed a meta-regression analysis using cognitive function (cognitive decline or dementia incidence) as the dependent variable and the length of follow-up as an independent variable in an adjusted model for potential confounders.

We tested heterogeneity among studies using the I^2^ test and by visual inspection of the forest plots. We interpreted I^2^ values of ≤25%, between 25% and 50%, and above 50% as “low,” “moderate,” and “high” heterogeneity between studies, respectively. We also computed the τ^2^ to assess the between-study variance and reported the 95% prediction intervals to evaluate the effect size variation of a future new study. In the nonlinear analysis, we also assessed the variation across individual study results, showing the study-specific trends using predicted curves [36]. We used Stata-MP version 18.0 (StataCorp LLC, 2023) for all statistical analyses, specifically the “meta,” “mkspline,” and “drmeta” routines.

### Quality assessment

We assessed the quality and risk of bias of the included studies with the Nutrition Quality Evaluation Strengthening Tools, specially developed for dietary methods assessment [37]. We used the version for cohort studies that consists of 4 domains related to the cohort selection, comparability, ascertainment of the outcomes, and nutrition specific. The overall rating is expressed as poor (most criteria are not met, leading to a high risk of bias), neutral (most criteria are met and are of little or no concern), and good (almost all criteria are met, leading to a low risk of bias). Study quality was evaluated by 2 reviewers (FV and NO), and discrepancies in each domain were resolved with the help of a third author (TF) in case of disagreements. We used Egger’s test and funnel plot to visually assess the indication of publication bias [38].

## Results

**FIGURE 1:**
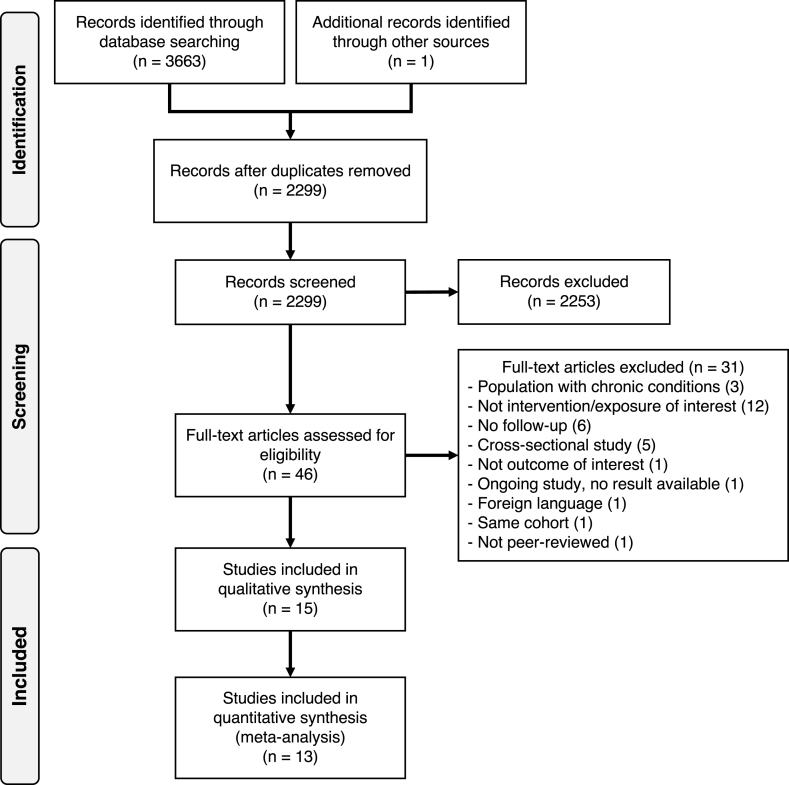
PRISMA flow diagram summarizing literature search, study identification and selection.

The systematic search identified 3663 records (Figure 1), and 1 additional article was retrieved through reference list scanning. After removing duplicates, we screened 2299 records, of which 2253 were excluded based on title and abstract screening. We retrieved 46 full-text articles for evaluation. We excluded 31 articles based on the eligibility criteria: population with chronic conditions (*n* = 3), not evaluating milk or dairy (*n* = 12), follow-up duration <6 mo (*n* = 6), cognitive decline or dementia not the outcome of interest (*n* = 1), no results available (*n* = 1), not in English language (*n* = 1), cross-sectional studies (*n* = 5), not peer-reviewed (*n* = 1), and same cohort as another included study (*n* = 1).

We included the remaining 15 studies, all with prospective cohort design and including a total of 312,580 participants (Table 1). Participants mean age ranged from 53 [17] to 91 y [16] at baseline. In the study by Yamada et al. 2003 [26] in the Adult Health Follow-Up study, participants were 30 y and older [26]. Seven studies were from Europe [16,18,25,[39], [40], [41], [42]], 6 studies from Asia [15,17,19,24,26,43], 1 from Australia [23], and 1 from the United States [44]. Participants were followed for a minimum of 4.8 y [23] to a maximum of 30 y [26] with a median follow-up of 11.4 y. Among the selected studies, 5 studies included the outcome of dementia incidence using International Classification of Diseases 8-10 or Diagnostic and Statistical Manual-IIIR/Diagnostic and Statistical Manual-IV criteria [16,18,26,39,43], and 10 studies evaluated cognitive function [15,17,19,[23], [24], [25],[40], [41], [42],^44^]. Most studies evaluated cognitive function with the Mini-Mental State Examination [16,17,19,[23], [24], [25],^41^], whereas others used other neuropsychological tests [[40], [41], [42],^44^]. Six studies used food frequency questionnaires [[15], [16], [17],^25^,^39^,^41^,^43^,^44^], including between 26 [16] and 188 [35] food items. Other studies used dietary records [18,24,40], dietary history [42], or other questionnaires [19,23,26]. Although 2 studies only evaluated milk intake (high fat [23] or total [44]) and 1 cheese intake [39], most studies evaluated total dairy intake [[15], [16], [17], [18], [19],^24^,^25^,[40], [41], [42],^45^]. The selection of covariates for adjustment was diverse; most studies adjusted their results for age, sex, education, physical activity, BMI, and previous comorbidities. Almost all studies adjusted their results for total calorie intake, except those without a full dietary assessment [16,19,23,26]. Moreover, some studies adjusted their outcomes for additional nutritional factors, for example, fruit/vegetable intakes [15,17,18,39] or “healthy” dietary patterns [17,40,43], among others.

**TABLE 1:** Summary of studies included in the meta-analysis

Author, year, cohort name, country	Follow-up (y)	Male (%)	Baseline age (y)	Number of participants	Exposure (method of assessment)	Dairy products dose (g/d)	Outcome (method of assessment)	Adjustments
Almeida, 2006 [23], NR, Australia	4.8	100	77.5	601	Consumed full-cream milk (self-reported questionnaire)	-	Cognitive function (MMSE, GDS-15)	Age, history of diabetes, consumption of full-cream milk, high school or university education, and vigorous physical activity
Dobreva, 2022 [39], UK Biobank, UK	11.4	46.7	62	249,511	Cheese intake (FFQ)	-	All-cause dementia (ICD 9 and 10)	Sociodemographic (age, sex, Townsend deprivation score, age left education, household income), lifestyle (physical activity, smoking status, weekly alcohol units), mental health factors (loneliness, depression), and physical health factors (BMI, cholesterol, diabetes, hypertension, cardiovascular events, major dietary changes) and all other food categories
Kesse-Guyot, 2016 [40], The SU.VI.Max 2 Observational Follow-Up Study, France	13	52	53.7	3076	Total dairy products (24-h dietary records)	-	Cognitive function (RI-48 test, verbal fluency tasks, digit span tests, and TMT)	Age, sex, education and follow-up time between baseline and cognitive evaluation, occupational status, intervention group during the trial phase, smoking status, physical activity, alcohol consumption, depressive symptoms, baseline memory troubles, BMI, energy intake, number of 24 h dietary records and history of diabetes, hypertension and CVD, Western and healthy dietary pattern score
Lu, 2023 [15], The Ohsaki Cohort 2006 Study, Japan	5.7	44.5	73.5	11,636	Total dairy intake (FFQ), milk, yogurt and cheese intake	Mean (SD) 116.8 (81.4) g/1000 kcal/d	Incidence of dementia (LTCI system based on Dementia Scale)	Sex, age, education level, BMI, smoking status, alcohol drinking status, time spent walking, psychological distress, history of diseases, energy intake, energy-adjusted vegetable and fruit intake, and energy-adjusted fish intake
Nicoli, 2021 [16], The Monzino 80-Plus Study, Italy	12	31	91.1	512	Milk and cheese intake (FFQ)	-	Incidence of dementia (DSM-IV)	Age, sex, education, total energy intake, smoking, alcohol, physical activity, chronic obstructive pulmonary disease, lifetime depression, previous stroke, previous transient ischemic attack, and place of residence
Otsuka, 2014 [24], National Institute for Longevity Sciences – Longitudinal Study of Aging, Japan	Male: 8.0; female: 8.2	51.6	Male: 67.7; female: 68.0	Male: 1137; female: 1065	Milk and dairy products (3-d dietary record)	Mean (SD) 164.77 (129.3)	Cognitive function (MMSE)	Age, follow-up time, MMSE score at baseline, education, BMI, household annual income, current smoking status, energy intake, and history of heart disease, hypertension, hyperlipidemia, and diabetes
Ozawa, 2014 [43], The Hisayama Study, Japan	17	42.3	69.4	1081	Milk and dairy consumption (FFQ)	Median (IQR) 97 (45–197)	All-cause dementia, AD, VaD (DSM-III)	Age, sex, low education, history of stroke hypertension, diabetes mellitus, total cholesterol, BMI, smoking habits, regular exercise and energy, vegetable, fruit, fish, and meat intake
Petruski-Ivleva, 2017 [44], ARIC (The Atherosclerosis Risk in Communities) Cohort, United States	20	44	57.5	13,752	Milk intake (FFQ)	Categorical	Cognitive function (DWRT, DSST, WFT)	Age, sex, race center, education level, APOE4, BMI, smoking, alcohol intake, diabetes, physical activity, total energy intake, diet quality
Talaei, 2020 [17], Singapore Chinese Health Study, Singapore	23	40.8	53	16,948	Dairy products (FFQ)	Median (IQR) (28.7 11.0–83.7)	Cognitive impairment (MMSE)	Age, sex, dialect, year of interview, educational level, marriage status, BMI, physical activity, smoking status, alcohol use, baseline history of self-reported hypertension, diabetes, heart attack, and stroke, history of cancer, sleep status, total energy intake, soy, red meat, poultry, fish, vegetables, fruits, tea, coffee, and soda, vegetable-fruit-soy dietary pattern
Tanaka, 2008 [41], InCHIANTI Study, Italy	Mean 10.1; max 18.2	43.5	75.4	832	Dairy products (FFQ)	Mean (SD) 170.3 (141.7)	Cognitive function (MMSE and additional neuropsychological tests)	Age, sex, study site, chronic diseases, years of education, total energy intake, physical activity, BMI, APOE4 carrier status, CRP, IL-6, plasma omega (ω)-3, plasma ω-6, plasma beta-carotene, and plasma alpha-tocopherol
Trichopoulou, 2015 [25], EPIC-Greece (European Prospective Investigation into Cancer and Nutrition-Greece), Greece	Median 6.8; range 5.1–8.2	35.9	74	401	Dairy products (FFQ)	Median (IQR) 205 (130–333)	Cognitive decline (MMSE)	Sex, age, years of education, BMI, physical activity, smoking, diabetes, hypertension, cohabiting, and total energy intake
Vercambre, 2009 [42], E3N (Etude Epidémiologique auprès de femmes la Mutuelle Générale de l’Education Nationale) Subcohort, France	13	0	65.5	4809	French Dietary History Questionnaire	Mean (SD) 283.6 (231.1)	Cognitive decline (DECO)	Age at cognitive assessment, education level, BMI, physical activity, energy intake, smoking status, use of supplements, use of postmenopausal hormones, depression, cancer, CHD, stroke, diabetes, hypertension, hypercholesterolemia
Yamada, 2003 [26], Adult Health Study Follow-Up Study, Japan	25	26.8	>30	1774	Milk intake (dietary questionnaire)	Categorical	AD, VaD (DSM-IIIR and DSM-IV)	Age, sex, education, and 10 mm Hg systolic blood pressure increase
Ylilauri, 2022 [18], Kuopio Ischemic Heart Disease Risk Factor Study, Finland	21.9	100	53	2416	Dairy products (4-d dietary records)	Mean (SD) [median] 711 (360) [688] 27% fermented	Any dementia, AD (ICD 8, 9 and 10)	Age, baseline examination year, energy intake, education years, pack-years of smoking, BMI, diabetes, leisure-time physical activity, history of coronary artery disease, use of lipid-lowering medication, intakes of alcohol, fiber, the sum of fruits, berries, and vegetables and dietary fat quality (ratio of PUFAs plus MUFAs to SFAs plus trans fatty acids)
Zhang, 2021 [19], Chinese Longitudinal Healthy Longevity Survey, China	6	50.7	77.8	3029	Dairy intake (frequency dietary questionnaire)	-	Cognitive decline (MMSE)	Sex, age, education, occupation before retirement, marital status, smoking, alcohol drinking, physical exercise, BMI, hypertension, diabetes, heart disease, and cerebrovascular disease

AD, Alzheimer’s disease; ApoE4, apolipoprotein E4; BMI, body mass index; CHD, chronic heart failure; CRP, C reactive protein; CVD, cardiovascular disease; DECO, détérioration cognitive observée; Dementia Scale, degree of independence in daily living for elderly with dementia; DSM, diagnostic and statistical manual; DSST, digit symbol substitution test; DWRT, delayed word recall test; FFQ, frequency food questionnaire; GDS, geriatric depression scale; ICD, International Classification of Disease; IL, interleukin; IQR, interquartile range; LTCI, long-term care insurance; MMSE, Mini-Mental State Examination; MUFA, monounsaturated fatty acid; NR, not reported; PUFA, polyunsaturated fatty acid; RI-48 test, Rappel indicé; SD, standard deviation; SFA, saturated fatty acid; TMT, trail making test; UK, United Kingdom; USA, United States of America; VaD, vascular dementia; WFT, word fluency test.

The assessment with the Nutrition Quality Evaluation Strengthening Tools revealed that out of 15 studies, there were 1 poor, 10 neutral (67%) and 4 good studies. Even if none of the studies assessed if the exposure difference was maintained over the study period, 14 out of 15 were rated as good in the nutrition domain. The main risk of bias came from the comparability domain because few of them reported the baseline differences between those lost to follow-up and the included participants, compared how many participants were lost to follow-up in each exposure group, or performed repeated measurements of the nutritional aspect under study. The detailed results are available in Supplementary Table 1.

**FIGURE 2:**
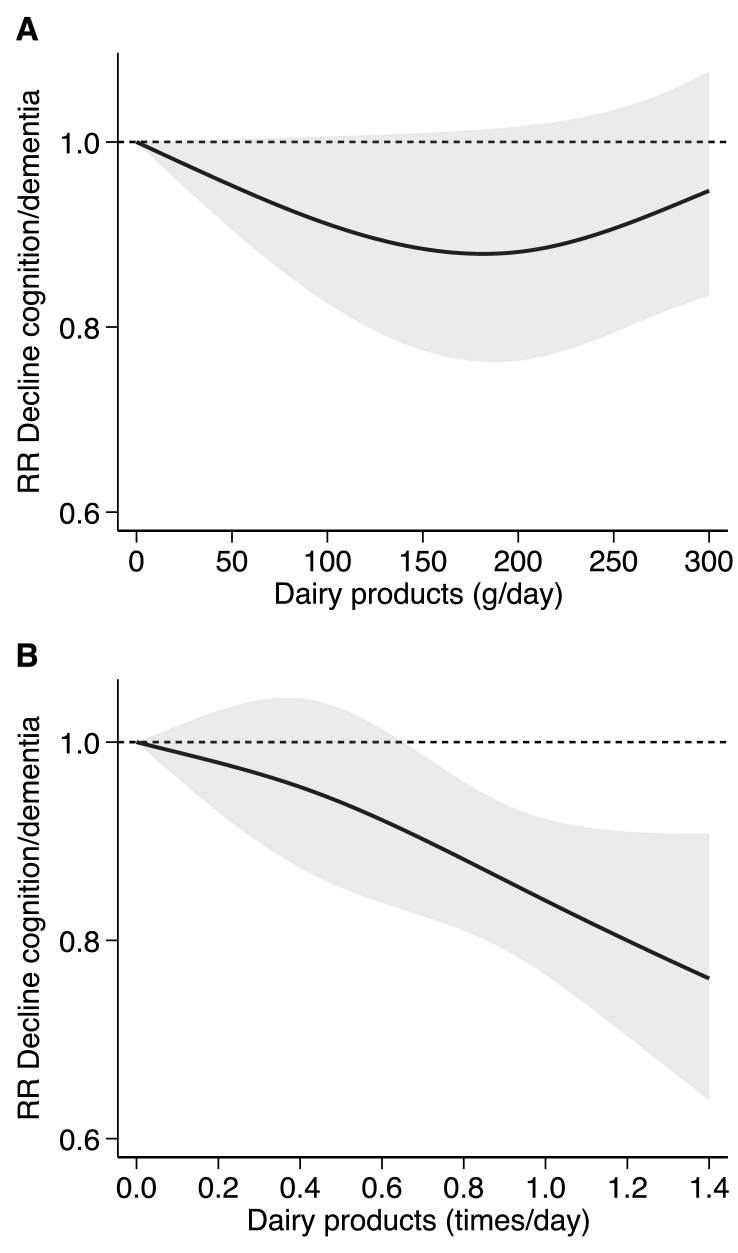
Dose-response analysis according to the quantity of consumption of dairy products in grams/day (A) 6 studies: Lu 2023 (Asia), Ozawa 2014 (Asia), Talaei 2020 (Asia), Tanaka 2018 (Europe), Vercambre 2009 (Europe), and Ylilauri 2022 (Europe); frequency of consumption of dairy products in times/day (B) 5 studies: Dobreva 2022 (Europe), Nicoli 2021 (Europe), Talaei 2020 (Asia), Yamada 2003 (Asia), Zhang 2021 (Asia). Spline curve (solid black line) with 95% confidence limits (gray area). RR: risk ratio.

The dose-response analyses (Figure 2) included 10 studies that had sufficient information on the consumption of dairy products by increasing quantity [15,17,18,[41], [42], [43]] or by increasing frequency [16,17,19,26,39] in relation to cognitive decline or dementia. When assessing the quantity of consumption, we observed a nonlinear association, with an initial decline in risk until 150 g/d (RR: 0.88; 95% CI: 0.78, 0.99), after which a slight change in direction was observed. We found an almost linear negative association when we considered the frequency of consumption (RR for linear trend 0.84; 95% CI: 0.77, 0.92 for 1 time/d increase of dairy products).

The results of the combined outcome (i.e., dementia or cognitive decline) showed that the highest intake of dairy products compared to the lowest intake has no association with cognitive decline or dementia (RR: 0.94; 95% CI: 0.82, 1.07) with high heterogeneity (I^2^: 69.2%) and between-study variance (τ^2^: 0.03) as showed by the wide prediction intervals (95% CI: 0.61, 1.45) (Supplementary Figure 1). For the outcome cognitive decline, we were able to combine 7 of the 9 studies [17,19,[23], [24], [25],^41^,^42^]: we observed no associations of the highest compared with the lowest dairy intake on cognitive decline (RR: 1.01; 95% CI: 0.86, 1.20) with high heterogeneity (I^2^: 73.5%) and between-study variance (τ^2^: 0.03) and wide prediction intervals (95% CI: 0.60, 1.72). Only 2 studies reported continuous results for cognitive function [40,44] and total dairy intake using linear regression analysis; thus, a meta-analysis with risk estimates was not possible. For the outcome of incident dementia, we identified 6 studies [15,16,18,26,39,43]. We observed a decreased risk of dementia with the highest intake of dairy compared with the lowest intake (RR: 0.83; 95% CI: 0.67, 1.03), although characterized by high heterogeneity (I^2^: 63.0%) and between-study variance (τ^2^: 0.04) leading to wide prediction intervals (95% CI: 0.44, 1.59) (Supplementary Figure 1).

In subgroup analyses, we observed that part of the heterogeneity could be explained by sex as studies carried out in both males and females reported inverse association (RR: 0.85; 95% CI: 0.78, 0.93) also characterized by negligible heterogeneity (I^2^: 2.6%, τ^2^: 0.00), whereas the studies reporting sex-specific results showed very heterogeneous and imprecise positive (in males) or null (in females) associations (Supplementary Figure 2). The dose-response meta-analysis restricted to such studies carried out in both sexes [15,17,41,43] showed a nonlinear association, although imprecise because of the lower number of studies, with a nadir at 100–150 g/d (Supplementary Figure 3).

Stratified analysis by age at recruitment of study participants showed lower risk in studies considering younger subjects <65 y (RR: 0.88; 95% CI: 0.76, 1.01) also characterized by limited heterogeneity (I^2^: 24.3%, τ^2^: 0.01) compared to studies recruiting older subjects ≥65 y (RR: 0.95; 95% CI: 0.75, 1.21, I^2^: 77.4%, τ^2^: 0.08) (Supplementary Figure 4).

**FIGURE 3:**
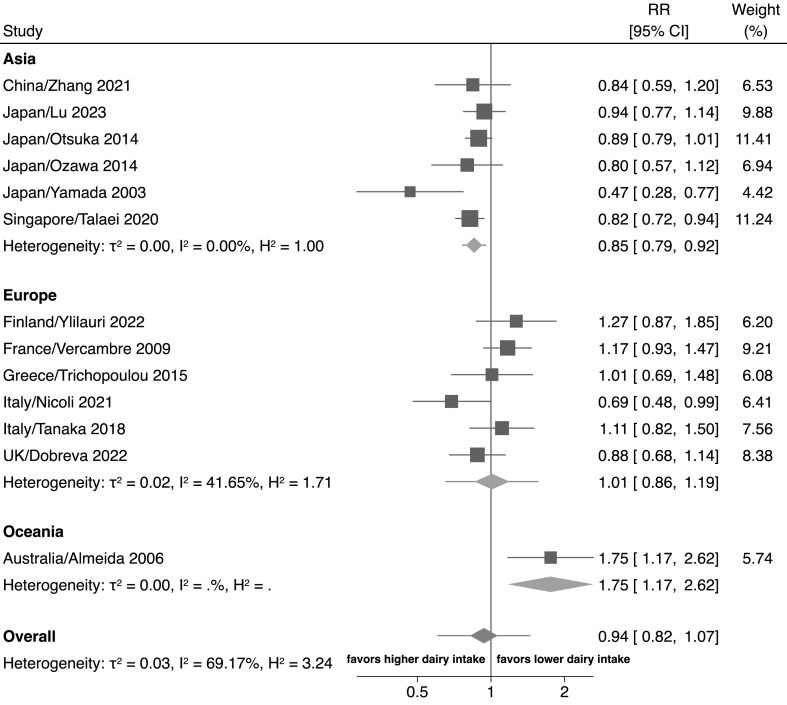
Forest plot showing the highest compared with lowest exposure meta-analysis of dairy intake and cognition divided by region. The area of each gray square is proportional to the inverse of the variance of the estimated log RR (i.e., weight in percentage) and the horizontal line the 95% CI of each individual study. Vertical axis of the gray diamonds represents the point estimate of the overall RR and the vertical axis its 95% CI, whereas horizontal line represents the 95% prediction interval intervals (CIs). The solid vertical line represents RR: 1. RR, risk ratio; CI, confidence interval.

In the subgroup analyses by region of origin (Figure 3), there was a reduced risk of cognitive decline or dementia with the highest dairy intake compared with the lowest dairy intake in the studies from Asia (RR: 0.83; 95% CI: 0.75, 0.92, I^2^: 0.0%, τ^2^: 0.00) [15,17,19,24,26,43]. Conversely, we found no association between dairy and cognitive decline or incident dementia among studies from Europe (RR: 1.01; 95% CI: 0.86, 1.19, I^2^: 41.6%, τ^2^: 0.02) [16,18,25,39,41,42] and higher risk with the highest intake compared with the lowest dairy intake in 1 single study from Oceania (RR: 1.75; 95% CI: 1.17, 2.62).

**FIGURE 4:**
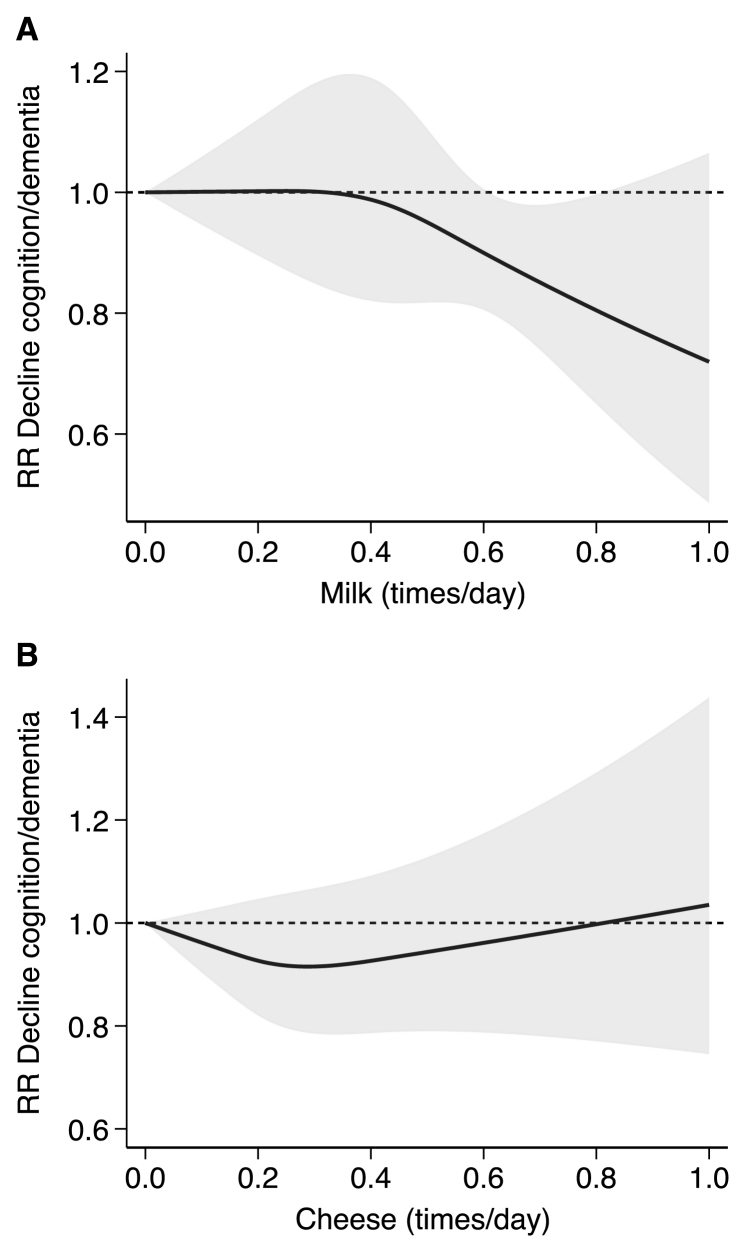
Dose-response analysis according to the frequency of consumption of dairy products in times/day divided by type of dairy product: milk reported in 3 studies: Lu 2023, Talaei 2020, and Yamada 2003 (A); and cheese reported in 2 studies: Dobreva 2022 and Lu 2023 (B). Spline curve (solid black line) with 95% confidence limits (gray area). RR, risk ratio.

In the analysis investigating different types of dairy products (Supplementary Figure 5), we found an inverse association with cognitive decline or dementia when all dairy types are considered (RR: 0.89; 95% CI: 0.83, 0.95, I^2^: 0.33%, τ^2^: 0.00). Conversely, the association with specific dairy products was very heterogeneous and inconsistent as it was reported in a lower number of studies, with the exception of milk and cheese intake alone, investigated in 5 and 4 studies and reporting both null associations, respectively. The dose-response meta-analysis by dairy type (Figure 4) was feasible for these latter subgroups. The analysis showed a null association with milk consumption ≤0.3 times/d, whereas a negative association emerged for high intakes. Conversely, the association seemed to be nonlinear for cheese consumption, with lower risk at 0.3 times/d and null/positive association at higher intakes.

The sensitivity analysis excluding the 1 study judged at possible high risk of bias [23] suggests a stronger negative association between dairy intake for cognitive decline or dementia outcome (overall RR: 0.90; 95% CI: 0.82, 1.00) with decreased heterogeneity (I^2^: 44.7%) and lower study variance (τ^2^: 0.01) despite the still wide prediction intervals (95% CI: 0.69, 1.18) (Supplementary Figure 6). In addition, the association became slightly negative for cognitive decline (RR: 0.94; 95% CI: 0.83, 1.07). Conversely, the dose-response meta-analysis did not change as the 1 study at high risk of bias was excluded, already not reporting exposure doses of dairy intake.

Stratified analysis by duration of follow-up (<10 y and ≥10 y) showed little influence on the overall estimate (Supplementary Figure 7). Similarly, the meta-regression analysis for increasing years of length of follow-up adjusting for potential cofounders based on previous stratified analyses (i.e., sex, age category at recruitment, and region of origin) showed almost negligible association with risk of cognitive decline or dementia incidence (beta regression coefficient: –0.005; 95% CI: –0.023 to 0.014) (Supplementary Figure 8).

Assessment of small-study bias showed low effects, with symmetry of funnel plot and low effect-based Egger’s test (slope: –0.17; 95% CI: –2.78 to 2.44) (Supplementary Figure 9). Assessment of study-specific curves showed higher variation in studies using quantity compared to frequency of consumption of dairy intake (Supplementary Figure 10) when considering overall dairy products. Conversely, stratified analysis by dairy types showed high variation in both studies measuring milk and cheese intake using frequency of consumption (Supplementary Figure 11).

## Discussion

This systematic review and meta-analysis identified 15 prospective observational studies involving >300,000 participants. Results suggest that dairy might be associated with a lower risk of cognitive decline or dementia but that there may be differences by sex, age, region of origin, level of intake, and type of dairy products. To our knowledge, we are the first study to evaluate dose-response relationships in a meta-analysis of dairy and cognition, suggesting a nonlinear relation with lower risk at ∼150 g/d of overall dairy intake. Our subgroup analyses suggest that this could mainly be explained by differences in the level of intake and type of dairy products. As a matter of that, the intake of dairy products greatly varies across the included studies, mainly depending on the region of origin. Considering only studies in Asia, the highest dairy intake was associated with a much-reduced risk of cognitive decline or dementia and low heterogeneity compared with European studies. Among European studies, there was no association between dairy intake and cognitive decline or dementia. In contrast, the single study conducted in Oceania reported a higher risk of cognitive decline with the highest dairy intake compared to the lowest, although such a study was deemed at high risk of bias, thus limiting the reliability of such results. Similar results were reported in the 2016 meta-analysis by Wu et al. [14], wherein the stratified analysis by race, studies conducted among Asians had a 43% lower risk of cognitive disorders with higher dairy intakes, whereas for those conducted in Caucasians, there was no association. Divergent results between Asian and European countries have also been reported for stroke [46]. The amount and types of dairy consumption between regions were considerably higher in studies carried out in European countries, with mean value between 170–711 g/d, than studies in Asian countries where total mean dairy intake ranged between 29–165 g/d. Despite the “Westernization” of Asian diets, populations in Asian countries, in general, still consume lower quantities of dairy products [47]. Also, in Asian countries, recommendations for dairy intake range between 1–4 servings/d, whereas in Europe, they are slightly higher at 2–4 servings/d [48], and milk is consumed more frequently than other dairy products [46,49].

Dairy is a heterogeneous food group including fermented or nonfermented foods and differing in nutrients such as fat and sodium. Stratified analysis by dairy type suggested an inverse linear relation when milk intake was considered only, whereas the shape of the association seemed to be nonlinear for cheese intake. In the study by Kesse-Guyot et al. [40], total dairy intake was not associated with any of the cognitive outcomes; milk intake was associated with worse verbal memory, and yogurt and cheese were associated with better verbal memory in some models. In particular, the study reported detrimental of dairy products effects on working memory performance at intakes higher than recommended, possibly supporting the U-shape association we noted in the dose-response meta-analysis. Unfortunately, we were not able to perform additional analyses for other dairy types because of a limited number of studies. It is noteworthy that in the 2 studies investigating the relation between dairy desserts, a detrimental association was found with 30% higher odds of cognitive decline [42] and lower scores for both working and verbal memory [40]. It should be noted that guidelines for dairy intake rarely include dairy desserts, being generally included in sweets products as they may contain high amounts of sugar [50,51]. Overall, these results suggest that the different types of dairy can have opposite effects on cognition. Dairy is also a heterogeneous food group regarding the fat content. We were not able to stratify results by the amount of fat in dairy products (full-fat compared with low-fat products). Two previous studies suggested that the fat content of milk might be associated with worse cognition [23,42]. In line with the results by Vercambre et al. 2009 [42] (France), where dairy desserts and ice cream were associated with worse cognition, in the study by Almeida et al. 2006 [23] (Australia), higher intakes of “full-cream dairy” were associated with worse mental health outcomes. The study by Petruvski-Ivleva et al. 2017 [44] (United States) reported that higher total milk intake was associated with greater cognitive decline, and whereas they did not report stratified results, ≤75% of participants reported skim/low-fat milk intake, in contrast to the 2 previous studies. Therefore, the role of high fat compared with low-fat dairy is still controversial and should be further evaluated.

Dairy products are rich in proteins, minerals, vitamins, and essential amino acids that have been directly or indirectly associated with cognitive function [52,53]. Previous studies have shown the beneficial effects of some dairy products, in particular fermented products, on cardiovascular disease or diabetes [10,[54], [55], [56]], which could be mediators of the associations between dairy intake and cognitive decline [57]. Fermented dairy products have anti-inflammatory components that can affect the risk of dementia [7,9,58,59]. However, the high fat content in some dairy products can affect cognition negatively through hyperinsulinemia, endothelial damage, oxidative stress, and inflammation [53,60,61]. In a study about fat intake at midlife and cognitive decline that did not qualify for our review (as it reported only fat intake from foods, but not food intakes), high saturated fat intake from milk products and spreads was associated with poorer cognitive outcomes and the results did not change after adjusting for several cardiovascular risk factors and diseases [61]. In addition, calcium content may greatly vary among different types of dairy products with possible effects on oxidative stress as both consumption of dairy products and calcium intake have been associated with higher glutathione peroxidase in the brain, suggesting possible protective mechanisms of such detrimental association [62].

Concomitantly, lower intake of dairy products could be associated with a specific dietary pattern, rich in plant-based foods and low in saturated fats, which have been shown to positively modulate the inflammatory and immune response and to decrease the risk of neurocognitive impairments and eventually the onset of dementia [63]. For instance, higher adherence to the Mediterranean diet was associated with a positive effect on cognitive decline [64]. The Japanese-style diet has been associated with a lower risk of cardiovascular disease, stroke, or heart disease mortality [65]. However, according to the 2016 Japanese National Health and Nutrition Survey, consumers of a nondairy diet were less likely to meet dietary requirements, whereas dairy consumers were more likely to exceed the recommendations for saturated fat [66]. In fact, studies that took into account other food groups or dietary patterns that could affect the relationship between dairy consumption and cognitive function found no associations [17,18,39,40,43,44].

In our search, we did not identify any RCT evaluating the effect of dairy on cognition, probably because of our strict inclusion criteria regarding dairy and cognitive assessments, as well as the duration of the intervention longer than 6 mo. Given that we present only results from observational studies, interpreting the results regarding cause and effect between dairy and cognition should be done carefully. Most of the studies adjusted for sex, age at recruitment, physical activity, smoking status, BMI, educational level, and past major cardiovascular events (stroke, coronary artery disease, and myocardial infarction) or related risk factors (hypertension, dyslipidemia). Some of them failed to adjust for total calorie intake [17,19,26], depression or psychological distress [17,24,25,41,42], and cancer [[15], [16], [17],^24^,^41^,^45^]. However, we cannot discard that the observed association is affected by residual confounding. In addition, dietary assessments were heterogeneous regarding the type of questionnaires used, definitions of dairy intake, and recall timeline. In addition, each study defined the outcome for cognition differently, which may be the main challenge when interpreting the results of our review. Many studies used nonspecific global screening tools, many of which could have demographic biases if they have not suitably validated in representative populations.

Regarding the optimal dairy intake that can be associated with greater cognitive health, our dose-response analysis for the continuous intake of dairy products suggests a nonlinear association with nadir at 150 g/d of dairy intake. For example, this would be equivalent to consuming 1 yogurt or 1 glass of milk/d, corresponding to 125–200 g/4.4–7 oz of yogurt or 200–250 mL/6.8–8.5 oz of milk/d according to Food-Based Dietary Guidelines in Europe [67]. This is in line with the mean dairy intake in Japan among milk consumers (∼160 g) [66] but lower than the mean intake in Europe, where 91.6% consume 2 or more dairy servings per week in older adults [68]. However, these results should be interpreted with caution. The included studies used a variety of categories of milk intake (“times per week,” “times per day,” “g/d,” “serving/d,” “high/low intake,” “tertiles,” etc.). Many studies did not report exact doses for “servings” and “time”; therefore, only a limited set of studies could be included in this analysis.

Because most studies reported only 1 measurement of diet, this might not reflect long-term consumption patterns. The lack of multiple dietary assessments hampered the evaluation of possible changes of time of dairy intake. Even though some studies suggest that the recall of past dairy intake may be more reliable because of stable consumption [69,70], more recent prospective studies assessing dairy product consumption over the life course are needed to evaluate dairy consumption changes. By including prospective studies of long duration, we aimed to include subjects whose diet was monitored long before cognition was assessed. However, we cannot discard differential measurement error because of the recall bias, as early symptomatology of cognitive decline could have affected the way people report their diet or their dietary choices [71]. Deteriorating cognition could also impact food selection or dietary behaviors. However, most of the studies have a low prevalence of cognitively impaired subjects [17,26,40] or excluded them in the analysis [18,19,24,25,41,43], and for most studies, there were many years between dietary and cognitive assessments in many studies. In our review, the stratified analysis by duration of follow-up showed only a slight reduction of risk of cognitive decline with the highest dairy intake in studies of >10 y of follow-up that was also consistent with the meta-regression analysis, suggesting a slightly negative association with increasing follow-up duration. In the future, biomarkers of dairy intake could help prevent recall errors and multiple assessments of dietary habits [72].

In this review, our focus was specifically on studies conducted in relatively healthy populations and for primary prevention of cognitive decline. Consequently, we deliberately excluded studies involving only patients with conditions such as diabetes, hypertension, and other chronic diseases. The association between hypertension [73], diabetes [74], or metabolic syndrome [75] and dementia has been extensively studied, and these conditions are considered to be modifiable risk factors for dementia in contemporary guidelines [5]. Healthcare professionals are actively encouraging patients to modify their lifestyles as part of their clinical management [76]. In the context of cognitive decline and dementia, dietary modifications among these patients are actually for secondary rather than primary prevention. Therefore, dietary recommendations to prevent dementia among patients with chronic diseases at high risk of dementia might be different than the recommendations to the general population. Considering that studies conducted among patients usually recruit from hospitals, it’s essential to acknowledge that hospitalization can impact dietary recall and potentially influence recent dietary habits. Thus, dietary questionnaires collected during or close to a hospital stay may not accurately represent an individual’s typical long-term dietary exposure. Most importantly, dietary modifications to prevent further consequences of other chronic conditions might lead to reverse causation.

As the prevalence of chronic disease is very high in Western populations such as the United States population, being in the order of >10% for diabetes, nearly 50% for hypertension, and 40% for metabolic syndrome [77], the results and the findings of our meta-analysis would not be automatically and directly applicable to a substantial part of the population, limiting the generalizability of our results. Future studies should evaluate in detail the role of dairy intake on cognition among people with comorbidities such as diabetes and other populations at high risk of dementia.

As strengths of our study, we included only prospective studies and planned several subgroup analyses to address the heterogeneous results of the previous literature. However, we acknowledged that some amount of heterogeneity was still present in stratified analyses, probably linked to the different types of dairy products or to the method of outcome assessment characterized by high variation across studies and countries. Compared to previous meta-analyses of prospective observational studies on dairy intake and cognitive decline, we additionally included 5 recent studies and 2 older studies that were not included in the 2 previous meta-analyses [13,14], with the opportunity to implement several stratified analyses showing the effect modification of sex, region of origin, and especially types of dairy products. Nonetheless, the number of studies in some of them was still limited, with consequent high heterogeneity. In addition, restricting our analysis to individuals without (known) chronic diseases would have limited the external validity of our findings but may have increased the internal validity by avoiding the risk of reverse causation linked to dietary advice in participants with chronic disease, thus reducing the risk of bias in exposure assessment.

Our exclusion criteria allow us to focus on the long-term effects of usual dairy intake and prevent potential recall bias. However, this led to not including RCTs as they were of too short duration. In addition, because of the small number of studies reporting continuous effects and stratified analyses by type of dairy, we could not conduct relevant stratified analyses.

In conclusion, the results from our systematic review and meta-analysis suggest a potential negative association of dairy intake on dementia, with regional differences. Future studies should evaluate the role of specific types of dairy products on cognition, focusing on potential differences in dairy types, intake levels, and population characteristics.

## Author contributions

The authors’ responsibilities were as follows – POC-B: designed the study with feedback from NR, MV, and CDG; DK-H: prepared the literature search; FV and TF: conducted the systematic review and selected of the articles, with feedback from POC-B; FV and NO: performed the risk of bias assessment with feedback from TF; FV and TF extracted data for analysis; TF: conducted all statistical analyses; FV, TF, and POC-B: interpreted the results with feedback from all authors and wrote the first draft of the manuscript; POC-B: had primary responsibility for final content, and all authors: read and approved the final manuscript.

## Conflict of interest

The authors report no conflicts of interest.

## Funding

This systematic review and meta-analysis is funded by the SNF-project grant 204967 “Prospective international study of dairy and inflammation on cognitive decline” (PI: PC-B), which also funds NO. TF and MV were supported by the grant “Dipartimenti di Eccellenza 2018–2022” to the Department of Biomedical, Metabolic and Neural Sciences, University of Modena and Reggio Emilia from the Italian Ministry of University and Research. TF is supported by grants PRIN 2022 (no. 2022MHMRPR) and PRIN 2022 PNRR (no. P20229KSXB) from the Italian Ministry of University and by grant FAR2023 from the University of Modena and Reggio Emilia.

## Data availability

Data described in the manuscript, code book, and analytic code will be made available upon request, pending application and approval of the corresponding author.

## Appendix A Supplementary data

Multimedia component1 Multimedia component1
