# Supplementary material for: Dairy Intake and Risk of Cognitive Decline and Dementia: A Systematic Review and Dose-Response Meta-Analysis of Prospective Studies
Source: Adv Nutr. 2023 Dec 1;15(1):100160. doi: 10.1016/j.advnut.2023.100160 (PMC10788406; doi:10.1016/j.advnut.2023.100160)

**Villoz Fanny et al. Dairy intake and risk of cognitive decline and dementia: A systematic review and dose-response meta-analysis of prospective studies – Supplementary Material**

# Supplementary Material 1: Full Search Strategy per Database

*Research questions:* **Does dairy intake prevent cognitive decline and reduce the incidence of dementia in the adult population?**

In databases where a controlled vocabulary was available, articles were searched by thesaurus terms (MeSH, Emtree) as well as free text terms in title and abstract, in other databases by free text terms only.

**Embase.com (Elsevier)**

| **1) Dairy Products (without breast milk)** |
| --- |
| (('dairy product'/exp OR (dairy OR milk OR milkfat* OR milkpowder* OR yogurt* OR yoghurt* OR yoghourt* OR joghurt* OR skyr OR kefir* OR koumiss* OR quark* OR butter OR buttermilk* OR butterfat* OR margarine* OR custard* OR cheese* OR fromage* OR cheddar* OR parmesan* OR muenster* OR curds OR mozzarella* OR ricotta* OR cream OR ghee OR whey* OR casein* OR lactalbumin*):ab,ti,kw) NOT (('breast milk'/exp OR ('breast milk' OR 'human milk' OR 'maternal milk' OR 'mother milk' OR 'woman milk' OR colostr*):ab,ti,kw) NOT ('dairy product'/exp OR ('bovine milk' OR 'cow* milk'):ab,ti,kw))) |
| **2) Cognitive decline, Dementia** |
| ('cognitive defect'/exp OR 'cognitive decline'/exp OR 'dementia'/exp OR 'degenerative disease'/de OR 'neurodegenerative dementia'/de OR 'memory disorder'/exp OR 'short term memory'/de OR (((cognition OR cognitive OR memory) NEAR/3 (disorder* OR defect* OR deficit* OR disabil* OR disable* OR dysfunction* OR impair* OR deterior* OR block* OR performance*)) OR 'response interference*' OR dement* OR Alzheimer* OR neurodegenerat* OR neuro-degenerat*):ab,ti,kw) |
| Exclusion: NOT ([animals]/lim NOT [humans]/lim) NOT ([Conference Abstract]/lim OR [Letter]/lim OR [Note]/lim OR [Editorial]/lim) |
| Inclusion: Adults (>18) only: NOT (([newborn]/lim OR [infant]/lim OR [child]/lim OR [adolescent]/lim) NOT [adult]/lim) |

**Embase.com (Elsevier)**

((('dairy product'/exp OR (dairy OR milk OR milkfat* OR milkpowder* OR yogurt* OR yoghurt* OR yoghourt* OR joghurt* OR skyr OR kefir* OR koumiss* OR quark* OR butter OR buttermilk* OR butterfat* OR margarine* OR custard* OR cheese* OR fromage* OR cheddar* OR parmesan* OR muenster* OR curds OR mozzarella* OR ricotta* OR cream OR ghee OR whey* OR casein* OR lactalbumin*):ab,ti,kw) NOT (('breast milk'/exp OR ('breast milk' OR 'human milk' OR 'maternal milk' OR 'mother milk' OR 'woman milk' OR colostr*):ab,ti,kw) NOT ('dairy product'/exp OR ('bovine milk' OR 'cow* milk'):ab,ti,kw))) AND ('cognitive defect'/exp OR 'cognitive decline'/exp OR 'dementia'/exp OR 'degenerative disease'/de OR 'neurodegenerative dementia'/de OR 'memory disorder'/exp OR 'short term memory'/de OR (((cognition OR cognitive OR memory) NEAR/3 (disorder* OR defect* OR deficit* OR disabil* OR disable* OR dysfunction* OR impair* OR deterior* OR degenerate* OR block* OR performance*)) OR 'response interference*' OR dement* OR Alzheimer* OR neurodegenerat* OR neuro-degenerat*):ab,ti,kw)) NOT ([animals]/lim NOT [humans]/lim) NOT (([newborn]/lim OR [infant]/lim OR [child]/lim OR [adolescent]/lim) NOT [adult]/lim) NOT ([Conference Abstract]/lim OR [Letter]/lim OR [Note]/lim OR [Editorial]/lim)

**Medline (Ovid)**

(((exp Dairy Products/ OR (dairy OR milk OR milkfat* OR milkpowder* OR yogurt* OR yoghurt* OR yoghourt* OR joghurt* OR skyr OR kefir* OR koumiss* OR quark* OR butter OR buttermilk* OR butterfat* OR margarine* OR custard* OR cheese* OR fromage* OR cheddar* OR parmesan* OR muenster* OR curds OR mozzarella* OR ricotta* OR cream OR ghee OR whey* OR casein* OR lactalbumin*).ab,ti,kw.) NOT ((Milk, Human/ OR (breast milk OR human milk OR maternal milk OR mother milk OR woman milk OR colostr*).ab,ti,kw.) NOT (exp Dairy Products/ OR (bovine milk OR cow* milk).ab,ti,kw.))) AND (exp Cognition Disorders/ OR exp Dementia/ OR Neurodegenerative Diseases**/** OR exp Memory Disorders/ OR Memory, Short-Term/ OR (((cognition OR cognitive OR memory) adj3 (disorder* OR defect* OR deficit* OR disabil* OR disable* OR dysfunction* OR impair* OR deterior* OR block* OR performance*)) OR response interference* OR dement* OR Alzheimer* OR neurodegenerat* OR neuro-degenerat*).ab,ti,kw.)) NOT (exp animals/ NOT humans/) NOT
(rats or mice or mouse).ti. NOT ((exp infant/ or exp child/ or adolescent/) not (exp adult/)) NOT (letter* OR news OR comment* OR editorial* OR congres*).pt.

**Cochrane Library (Wiley)**

Cochrane Database of Systematic Reviews (2 results)
Issue 12 of 12, December 2022

Cochrane Central Register of Controlled Trials (277 results)
Issue 12 of 12, December 2022

((dairy OR milk OR milkfat* OR milkpowder* OR yogurt* OR yoghurt* OR yoghourt* OR joghurt* OR skyr OR kefir* OR koumiss* OR quark* OR butter OR buttermilk* OR butterfat* OR margarine* OR custard* OR cheese* OR fromage* OR cheddar* OR parmesan* OR muenster* OR curds OR mozzarella* OR ricotta* OR cream OR ghee OR whey* OR casein* OR lactalbumin*):ab,ti,kw NOT ("breast milk" OR "human milk" OR "maternal milk" OR "mother milk" OR "woman milk" OR colostr*):ab,ti,kw) AND ((((cognition OR cognitive OR memory) NEAR/3 (disorder* OR defect* OR deficit* OR disabil* OR disable* OR dysfunction* OR impair* OR deterior* OR block* OR performance*)) OR response NEXT interference* OR dement* OR Alzheimer* OR neurodegenerat* OR neuro-degenerat*****):ab,ti,kw)

*Additional information: sources of the RCTs found in CENTRAL (64 trials harvested from ClinicalTrials.gov, 67 from WHO ICTRP, 91 from Embase & 84 from PubMed)*

**Web of Science Core Collection (Clarivate)**

**(**Science Citation Index Expanded (SCI-EXPANDED), Social Sciences Citation Index (SSCI), Arts & Humanities Citation Index (A&HCI), Conference Proceedings Citation Index – Science (CPCI-S), Conference Proceedings Citation Index – Social Science & Humanities (CPCI-SSH))

TS=(((dairy OR milk OR milkfat* OR milkpowder* OR yogurt* OR yoghurt* OR yoghourt* OR joghurt* OR skyr OR kefir* OR koumiss* OR quark* OR butter OR buttermilk* OR butterfat* OR margarine* OR custard* OR cheese* OR fromage* OR cheddar* OR parmesan* OR muenster* OR curds OR mozzarella* OR ricotta* OR cream OR ghee OR whey* OR casein* OR lactalbumin*) NOT ("breast milk" OR "human milk" OR "maternal milk" OR "mother milk" OR "woman milk" OR colostr*)) AND ((((cognition OR cognitive OR memory) NEAR/2 (disorder* OR defect* OR deficit* OR disabil* OR disable* OR dysfunction* OR impair* OR deterior* OR block* OR performance*)) OR "response interference*" OR dement* OR Alzheimer* OR neurodegenerat* OR neuro-degenerat*))) NOT TS=((animal* OR plant* OR rats OR mice OR pigs) NOT (human* OR patient*)) AND DT=article

**Google Scholar (via Publish or Perish)** (first 200 results, out of 21’300)

dairy|milk|yogurt|joghurt|butter|buttermilk|margarine|cheese|cream|ice cream|casein|lactalbumin -"breast milk" cognition|cognitive|memory disorder|defect|deficit|disability|dysfunction|impairment|deterioration|performance dementia | Alzheimer | neurodegenerative -mice -rats

**Supplementary Material 2:** Full-text exclusion and reasons why

**Actrn, 2018**

- Actrn, *The Moo'D Study: a randomised controlled trial of A2 vs conventional dairy products in women with low mood.* <http://www.who.int/trialsearch/Trial2.aspx?TrialID=ACTRN12618002023235>, 2018.

*Reason for exclusion: Ongoing study, no result available*

**Anderson, 2018**

- Anderson, J.R., et al., *Baseline glucoregulatory function moderates the effect of dairy milk and fruit juice on postprandial cognition in healthy young adults.* European Journal of Nutrition, 2018. **57**(7): p. 2343-2352.

*Reason for exclusion: Measurment of outcome after of max 2 hours of exposure. Standard and running memory continuous preformance assessment after 30,90 and 120 min post-ingestion of milk, apple juice or water*

**Araki, 2017**

- Araki, A., et al., *Low intakes of carotene, vitamin B2, pantothenate and calcium predict cognitive decline among elderly patients with diabetes mellitus: The Japanese Elderly Diabetes Intervention Trial.* Geriatrics and Gerontology International, 2017. **17**(8): p. 1168-1175.

*Reason for exclusion: Population with chronic conditions*

**Bajerska, 2014**

- Bajerska, J., et al., *Eating patterns are associated with cognitive function in the elderly at risk of metabolic syndrome from rural areas.* European Review for Medical and Pharmacological Sciences, 2014. **18**(21): p. 3234-3245.

*Reason for exclusion: Missing Exposure of interest: Mediterranean diet*

**Chua, 2020**

- Chua, D., et al., *Dairy and fish consumption and the risk of mild cognitive impairment and dementia*. 2020: researchsquare.com

*Reason for exclusion: not peer-reviewed*

**Crichton, 2012**

- Crichton, G.E., et al., *Dairy consumption and working memory performance in overweight and obese adults.* Appetite, 2012. **59**(1): p. 34-40.

*Reason for exclusion: Measurement of outcomes only 6 months after exposure.*

**Crichton, 2012**

- Crichton, G.E., et al., *Relation between dairy food intake and cognitive function: The Maine-Syracuse Longitudinal Study.* International Dairy Journal, 2012. **22**(1): p. 15-23.

*Reason for exclusion: cross-sectionnal study*

**Flicker, 2005**

- Flicker, L., et al., *Predictors of impaired cognitive function in men over the age of 80 years: results from the Health in Men Study*. Age Ageing, 2005. **34**(1): p. 77-80.

*Reason for exclusion: same cohort than Almeida et al., 2006*

**Fournier, 2007**

- Fournier, L.R., et al., *The effects of soy milk and isoflavone supplements on cognitive performance in healthy, postmenopausal women.* Journal of Nutrition, Health and Aging, 2007. **11**(2): p. 155-164.

*Reason for exclusion: Cognitive functioning was assessed before and after the intervention*

**Gelber, 2012**

- Gelber, R.P., et al., *Lifestyle and the risk of dementia in Japanese-American men.* Journal of the American Geriatrics Society, 2012. **60**(1): p. 118-123.

*Reason for exclusion: Missing exposure of interest: diet pattern*

**Kanarek, 1990**

- Kanarek, R.B. and D. Swinney, *Effects of food snacks on cognitive performance in male college students.* Appetite, 1990. **14**(1): p. 15-27.

*Reason for exclusion: Measurement of outcomes after max 7h30 of exposure*

**Kim, 2004**

- Kim, J.H., Y. Lee, and G. Han, *Association of dietary factors with cognitive impairment in older women.* Journal of Preventive Medicine & Public Health / Yebang Uihakhoe Chi, 2004. **37**(2): p. 174-81.

*Reason for exclusion: Foreign language*

**Kim, 2018**

- Kim, K.Y. and J.M. Yun, *Association between diets and mild cognitive impairment in adults aged 50 years or older.* Nutrition Research & Practice, 2018. **12**(5): p. 415-425.

*Reason for exclusion: cross-sectionnal study*

**Klinedinst, 2020**

- Klinedinst, B.S., et al., *Genetic Factors of Alzheimer’s Disease Modulate How Diet is Associated with Long-term Cognitive Trajectories – A UK Biobank Study*. J Alzheimers Dis. 2020. 78(3): p.1245–1257

*Reason for exclusion: no follow-up data, only cross-sectional analysis of baseline*

**Lehtisalo, 2019**

- Lehtisalo, J., et al., Dietary changes and cognition over 2 years within a multidomain intervention trial—The Finnish Geriatric Intervention Study to Prevent Cognitive Impairment and Disability (FINGER). Alzheimer Dem, 2019. **15**(3): p. 410-417

*Reason for exclusion: Missing intervention of interest*

**Lu, 2022**

- Lu, Y., Matsuyama, S., Sugawara, Y., Sone, T., Tsuji, I*., Dairy intake and incident functional disability among older Japanese adults: the Ohsaki Cohort 2006 Study*. European Journal of Nutrition, 2022. 61(5). 2627-2637.

*Reason for exclusion: Missing outcome of interest, cognitive decline or dementia not assessed*

**Naghan, 2018**

- Naghan, P.A., et al., *The effect of doogh (yogurt drink) on reaction time and vigilance-sleepiness of healthy young adults.* Iranian Journal of Psychiatry and Behavioral Sciences, 2018. **12**(1).

*Reason for exclusion: Measurement of outcomes after 1.5 to 2 hours after exposure*

**Ni, 2022**

- Ni, J., et al., *Dairy Product Consumption and Changes in Cognitive Performance: Two-Year Analysis of the PREDIMED-Plus Cohort.* Molecular Nutrition & Food Research, 2022. **14**: p. 13.

*Reason for exclusion: Population with chronic condition*

**Ozawa, 2013**

- Ozawa, M., et al., *Dietary patterns and risk of dementia in an elderly Japanese population: The hisayama Study1-3.* American Journal of Clinical Nutrition, 2013. **97**(5): p. 1076-1082.

*Reason for exclusion: Missing exposure of interest: food groups and dietary pattern*

**Pawar, 2018**

- Pawar, M. and P. Magdum, Clinical study of assessment of therapeutic potential of vachadi ghrita, a medicated ghee formulation on healthy individual's cognition. International Journal of Pharmaceutical Sciences and Research, 2018. 9(8): p. 3408-3413.

*Reason for exclusion: Assessment of outcomes after 31 days of post-exposure*

**Prinelli, 2019**

- Prinelli, F., et al., *The impact of nutrient-based dietary patterns on cognitive decline in older adults.* Clinical Nutrition, 2019. **38**(6): p. 2813-2820.

*Reason for exclusion: Missing exposure of interest: Dairy-derived nutrients*

**Rahman, 2007**

- Rahman, A., et al., *Dietary factors and cognitive impairment in community-dwelling elderly.* Journal of Nutrition, Health and Aging, 2007. **11**(1): p. 49-54.

*Reason for exclusion: Cross sectional study*

**Shakersain, 2018**

- Shakersain, B., et al., *The Nordic prudent diet reduces risk of cognitive decline in the Swedish older adults: a population-based cohort study.* Nutrients, 2018.

*Reason for exclusion: Missing exposure of interest: food groups*

**Talhaoui 2023**

- Talhaoui, A., et al., *The relationship between adherence to a mediterranean diet and cognitive impairment among the elderly in Morocco*. Acta Neuropsychologica 2023 21:2 (125-138)

*Reason for excluson: cross sectional study*

**Tessier 2021**

- Tessier, A.J. et al., *Milk, Yogurt, and Cheese Intake Is Positively Associated With Cognitive Executive Functions in Older Adults of the Canadian Longitudinal Study on Aging*. Journals of Gerontology Series A-Biological Sciences & Medical Sciences - Volume 76, Issue 12, pp. 2223-2231.

*Reason for exclusion: cross-sectionnal study*

**Tomata, 2016**

- Tomata, Y., et al., *Dietary Patterns and Incident Dementia in Elderly Japanese: The Ohsaki Cohort 2006 Study.* The journals of gerontology. Series A, Biological sciences and medical sciences, 2016. **71**(10): p. 1322-1328.

*Reason for exclusion: Missing exposure of interest: Dietary pattern*

**Xu, 2018**

- Xu, X.Y., et al., *Dietary Pattern, Hypertension and Cognitive Function in an Older Population: 10-Year Longitudinal Survey.* Frontiers in Public Health, 2018. **6**: p. 13.

*Reason for exclusion: Missing exposure of interest: Dietary pattern*

**Yoshida, 2019**

- Yoshida, D., et al., *Dairy consumption and risk of functional disability in an elderly Japanese population: the Hisayama Study.* American Journal of Clinical Nutrition, 2019. **109**(6): p. 1664-1671.

*Reason for exclusion: Population with chronic conditions*

**Wei, 2022**

- Wei, K, et al., *Dietary Habits Modify the Association of Physical Exercise with Cognitive Impairment in Community-Dwelling Older Adults.* Journal of Clinical Medicine, 2022. **11**(17): p 30.

*Reason for exclusion: Missing exposure of interest*

**Zhang, 2009**

- Zhang, Z.X., et al., *Lifespan influences on mid- to late-life cognitive function in a Chinese birth cohort. Neurology, 2009. 73(3): p. 186-94.*

*Reason for exclusion: Missing exposure of interest. Milk consumption during childhood*

**Zhang, 2021**

- Zhang, Z., et al., Genetically Predicted Milk Intake and Risk of Neurodegenerative Diseases. Nutrients, 2021. 13: p. 2893.

*Reason for exclusion: Missing exposure of interest. Milk consumption using Mendelian randomization*

**Supplementary Table 1**. Risk of bias with Nutrition QUality Evaluation Strengthening Tools (NUQUEST) ratings for individual sections and overall

| **Study** | **Selection^2^** | **Comparability^3^** | **Ascertainment^4^** | **Nutrition^5^** | **Overall^6^** |
| --- | --- | --- | --- | --- | --- |
| Almeida 2006 (23) |  |  |  | 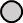 |  |
| Dobreva 2022 (37) | 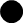 | 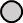 |  |  |  |
| Kesse-Guyot 2016 (38) |  | 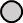 | 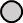 | 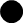 | 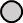 |
| Lu 2023 (15) | 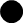 | 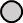 | 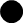 | 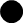 | 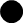 |
| Nicoli 2021 (16) | 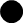 |  | 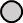 | 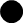 | 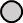 |
| Otsuka 2014 (24) | 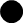 |  | 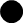 | 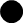 | 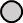 |
| Ozawa 2014 (41) | 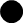 | 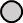 | 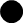 | 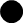 | 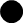 |
| Petruski-Ivleva 2017 (42) | 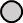 | 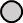 | 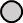 | 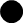 | 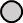 |
| Talaei 2020 (17) | 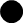 |  | 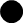 | 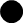 | 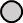 |
| Tanaka 2018 (39) | 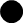 |  | 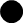 | 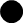 | 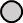 |
| Trichopoulou 2015 (25) |  |  | 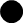 | 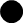 | 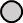 |
| Vercambre 2009 (40) | 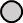 |  | 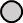 | 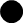 | 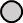 |
| Yamada 2003 (26) |  |  | 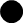 | 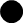 | 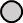 |
| Ylilauri 2022 (18) | 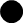 | 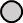 | 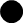 | 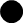 | 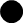 |
| Zhang 2021 (19) | 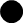 |  | 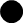 | 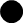 | 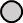 |
| ^1^ NUQUEST assessments are by section and overall, by study: *good*, where almost all criteria are met, little or no concern, and low RoB; *neutral*, where most criteria are met, there are some flaws, and moderate RoB; and  *poor*, where either most or all criteria are not met, there are significant flaws, and high RoB.  ^2^Selection = selection of cohorts. ^3^Comparability = comparability of cohorts. ^4^Ascertainment = ascertainment of outcomes. ^5^Nutrition = nutrition specific. ^6^Overall = overall NUQUEST rating. | | | | | |

Shannon E Kelly and others, NUQUEST—NUtrition QUality Evaluation Strengthening Tools: development of tools for the evaluation of risk of bias in nutrition studies, *The American Journal of Clinical Nutrition*, Volume 115, Issue 1, January 2022, Pages 256–271, <https://doi.org/10.1093/ajcn/nqab335>

**Supplementary Figure 1**. Forest plot showing the highest versus lowest exposure meta-analysis of dairy intake and cognitive function (overall and stratified by cognitive decline and dementia incidence). RR: relative risk; CI: confidence interval. The area of each gray square is proportional to the inverse of the variance of the estimated log RR (i.e. weight in %) and the horizontal line the 95% CI of each individual study. Vertical axis of the gray diamonds represents the point estimate of the overall RR and the vertical axis its 95% CI, while horizontal line represents the 95% prediction interval intervals (CIs). The solid vertical line represents RR=1.

**Supplementary Figure 2A**. Forest plot showing the highest versus lowest exposure meta-analysis of dairy intake and cognition divided by sex. RR: relative risk; CI: confidence interval. The area of each gray square is proportional to the inverse of the variance of the estimated log RR (i.e. weight in %) and the horizontal line the 95% CI of each individual study. Vertical axis of the gray diamonds represents the point estimate of the overall RR and the vertical axis its 95% CI. The solid vertical line represents RR=1.

**Supplementary Figure 2B**. Forest plot showing the highest versus lowest exposure meta-analysis of dairy intake and cognition divided by sex. RR: relative risk; CI: confidence interval. The area of each gray square is proportional to the inverse of the variance of the estimated log RR (i.e. weight in %) and the horizontal line the 95% CI of each individual study. Vertical axis of the gray diamonds represents the point estimate of the overall RR and the vertical axis its 95% CI, while horizontal line represents the 95% prediction interval intervals (CIs). The solid vertical line represents RR=1.

**Supplementary Figure 3.** Dose-response analysis according quantity of consumption of dairy products in g/day limited to studies carried out in both sexes: Lu 2023, Ozawa 2024, Talaei 2020 and Tanaka 2018). Spline curve (solid black line) with 95% confidence limits (grey area). RR: relative risk.


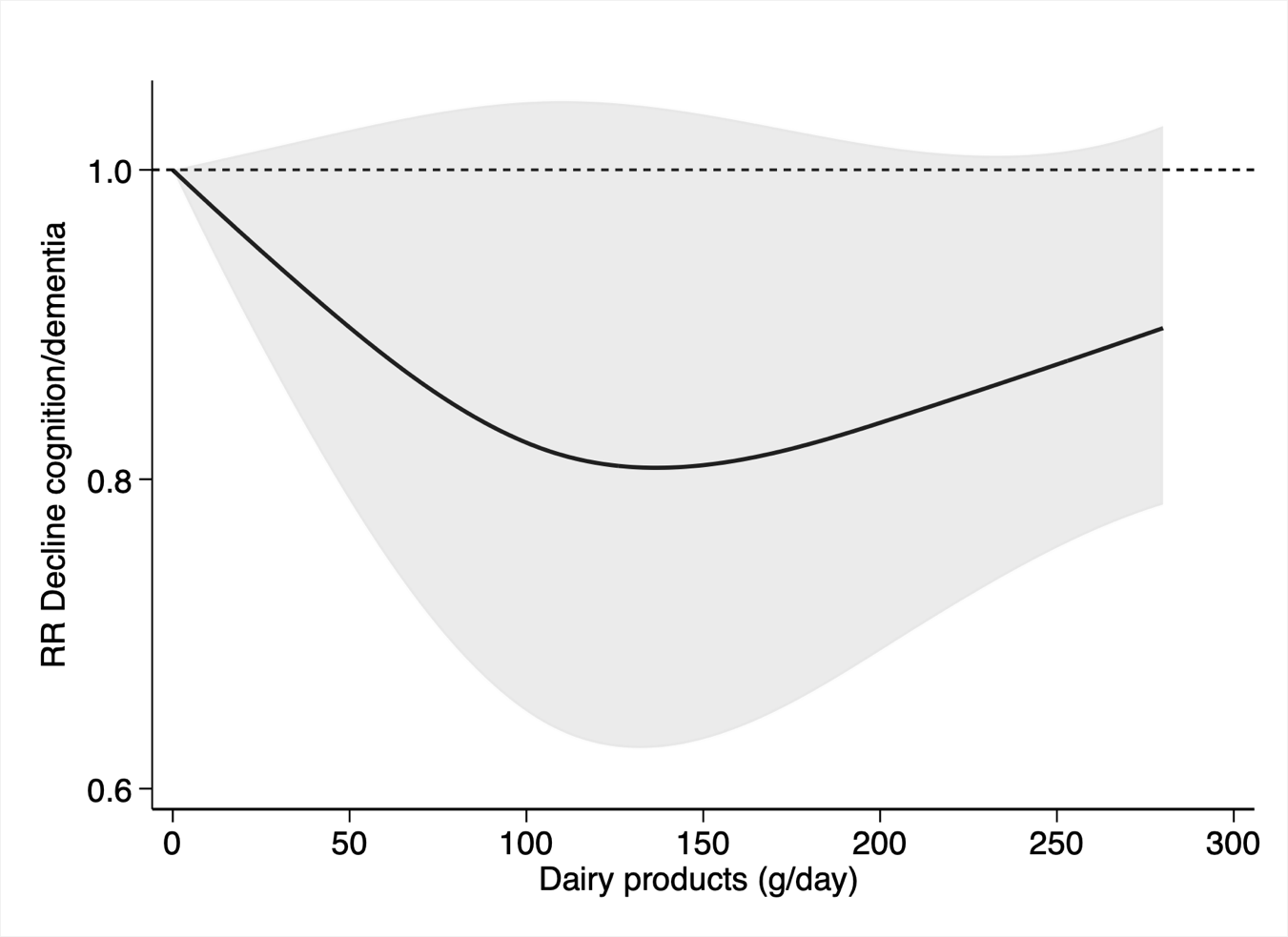


**Supplementary Figure 4**. Forest plot showing the highest versus lowest exposure meta-analysis of dairy intake and cognition divided by age at recruitment. RR: relative risk; CI: confidence interval. The area of each gray square is proportional to the inverse of the variance of the estimated log RR (i.e. weight in %) and the horizontal line the 95% CI of each individual study. Vertical axis of the gray diamonds represents the point estimate of the overall RR and the vertical axis its 95% CI, while horizontal line represents the 95% prediction interval intervals (CIs). The solid vertical line represents RR=1.

**Supplementary Figure 5**. Forest plot showing the highest versus lowest exposure meta-analysis of dairy intake and cognition divided by type of dairy. RR: relative risk; CI: confidence interval. The area of each gray square is proportional to the inverse of the variance of the estimated log RR (i.e. weight in %) and the horizontal line the 95% CI of each individual study. Vertical axis of the gray diamonds represents the point estimate of the overall RR and the vertical axis its 95% CI, while horizontal line represents the 95% prediction interval intervals (CIs). The solid vertical line represents RR=1.

**Supplementary Figure 6**. Forest plot showing the highest versus lowest exposure meta-analysis of dairy intake and cognitive function (overall and stratified by cognitive decline and dementia incidence) after exclusion of one study at high risk of bias. RR: relative risk; CI: confidence interval. The area of each gray square is proportional to the inverse of the variance of the estimated log RR (i.e. weight in %) and the horizontal line the 95% CI of each individual study. Vertical axis of the gray diamonds represents the point estimate of the overall RR and the vertical axis its 95% CI, while horizontal line represents the 95% prediction interval intervals (CIs). The solid vertical line represents RR=1.

**Supplementary Figure 7**. Forest plot showing the highest versus lowest exposure meta-analysis of dairy intake and cognition divided by duration of follow-up. RR: relative risk; CI: confidence interval. The area of each gray square is proportional to the inverse of the variance of the estimated log RR (i.e. weight in %) and the horizontal line the 95% CI of each individual study. Vertical axis of the gray diamonds represents the point estimate of the overall RR and the vertical axis its 95% CI, while horizontal line represents the 95% prediction interval intervals (CIs). The solid vertical line represents RR=1.

**Supplementary Figure 8**. Bubble-plot showing the meta-regression analysis between dairy intake and cognitive function (cognitive decline or dementia incidence) at increasing duration of follow-up in years, adjusted by sex, age at recruitment and region. The black solid line fits data from different studies and the shadowed area indicates 95% CI. Each hole circle represent one individual study with size proportional to the weight computed using the inverse-variance method.

**
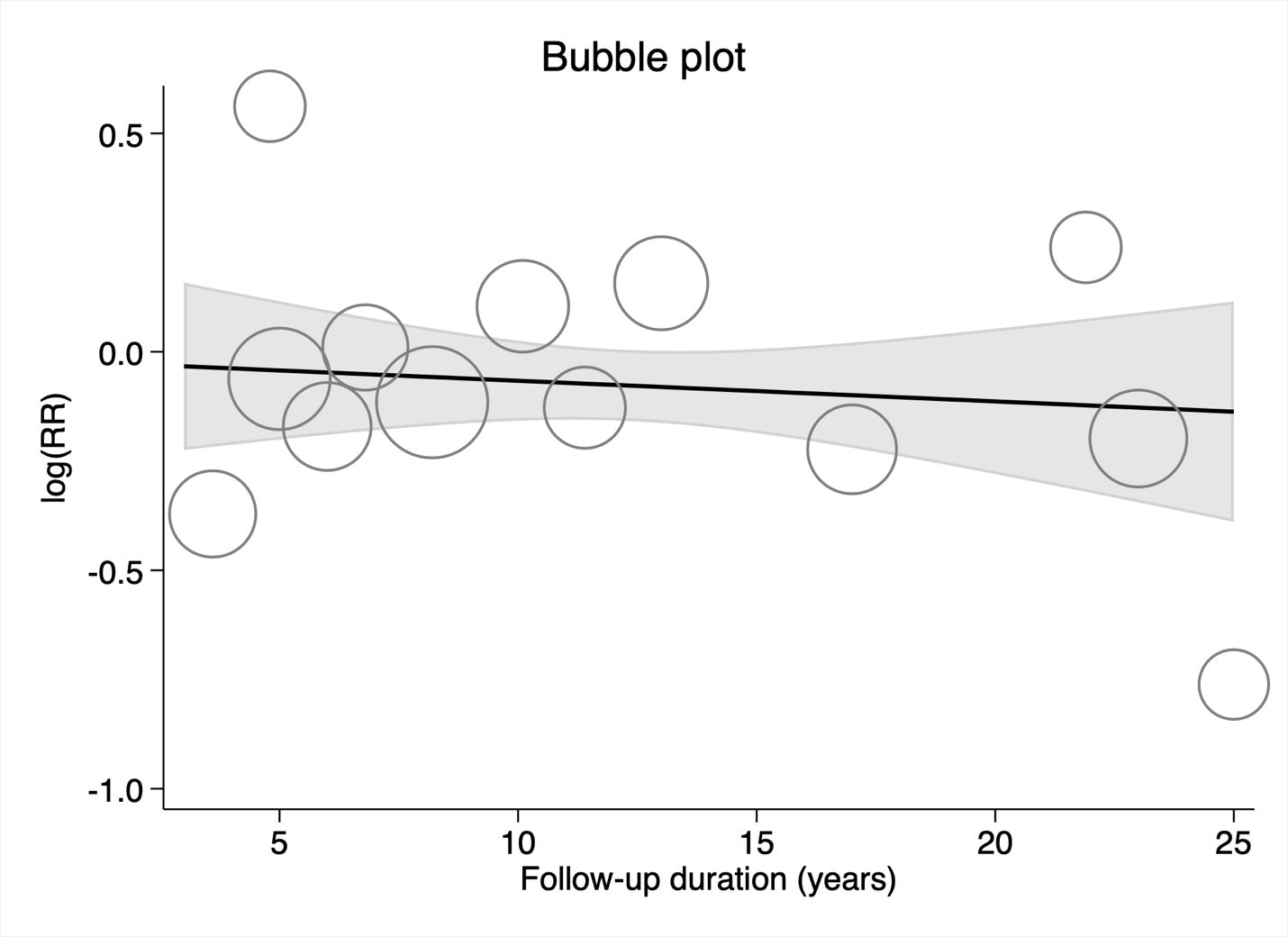
**

**Supplementary Figure 9**. Funnel plot for small-study bias of included studies.

**Supplementary Figure 10.** Dose-response analysis according: quantity of consumption of dairy products in g/day (A): six studies: Lu 2023 (Asia), Ozawa 2014 (Asia), Talaei 2020 (Asia), Tanaka 2018 (Europe), Vercambre 2009 (Europe) and Ylilauri 2022 (Europe); frequency of consumption of dairy products in times/day (B): five studies: Dobreva 2022 (Europe), Nicoli 2021 (Europe), Talaei 2020 (Asia), Yamada 2003 (Asia), Zhang 2021 (Asia). Spline curve (solid black line) with 95% confidence limits (grey area) for overall estimate and study specific-curves (dark gray lines) for individual study trends. RR: relative risk.


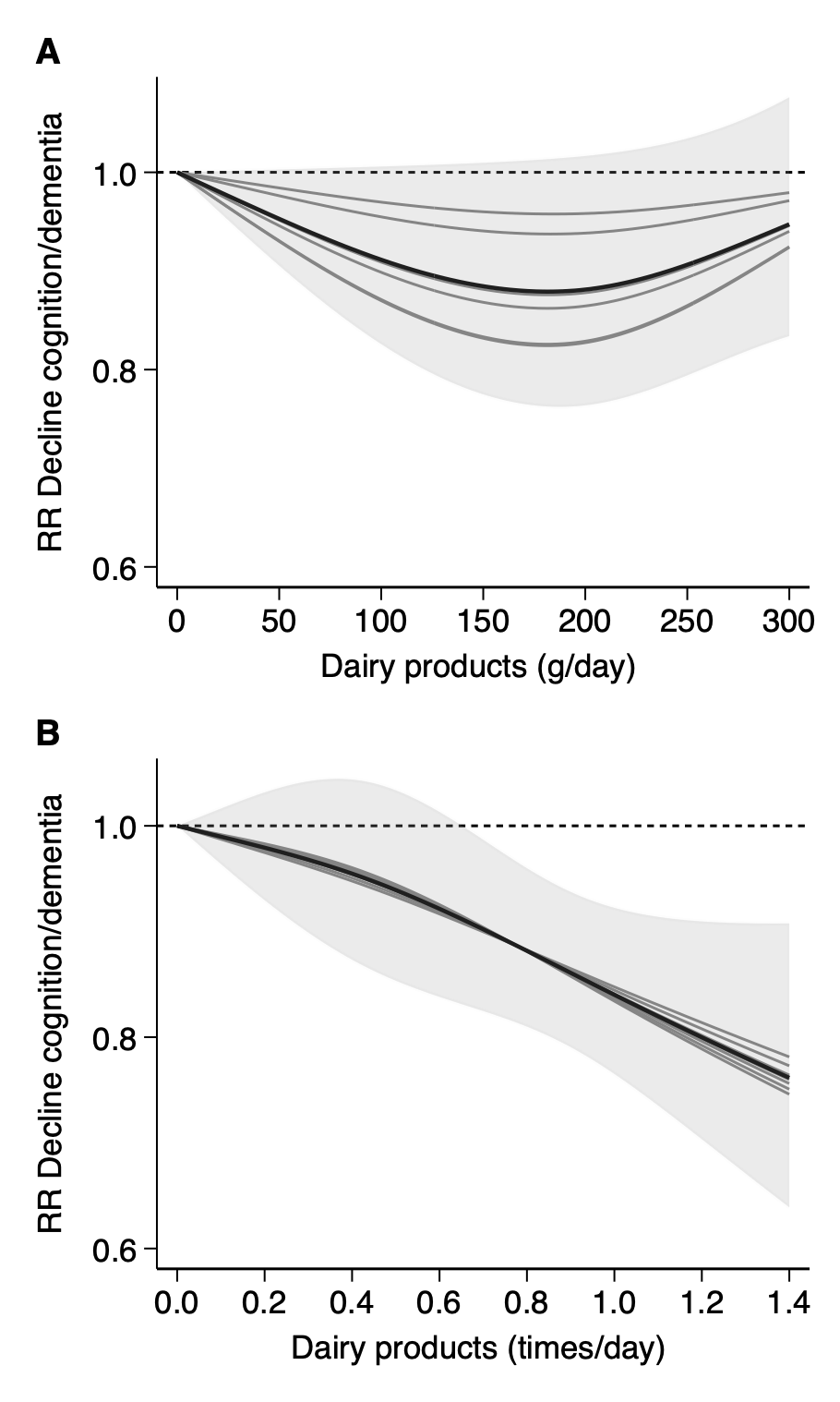


**Supplementary Figure 11.** Dose-response analysis according frequency of consumption of dairy products in times/day divided by type of dairy product: milk reported in three studies: Lu 2023, Talaei 2020, and Yamada 2003 (A); and cheese reported in two studies: Dobreva 2022 and Lu 2023 (B). Spline curve (solid black line) with 95% confidence limits (grey area) for overall estimate and study specific-curves (dark gray lines) for individual study trends. RR: relative risk.


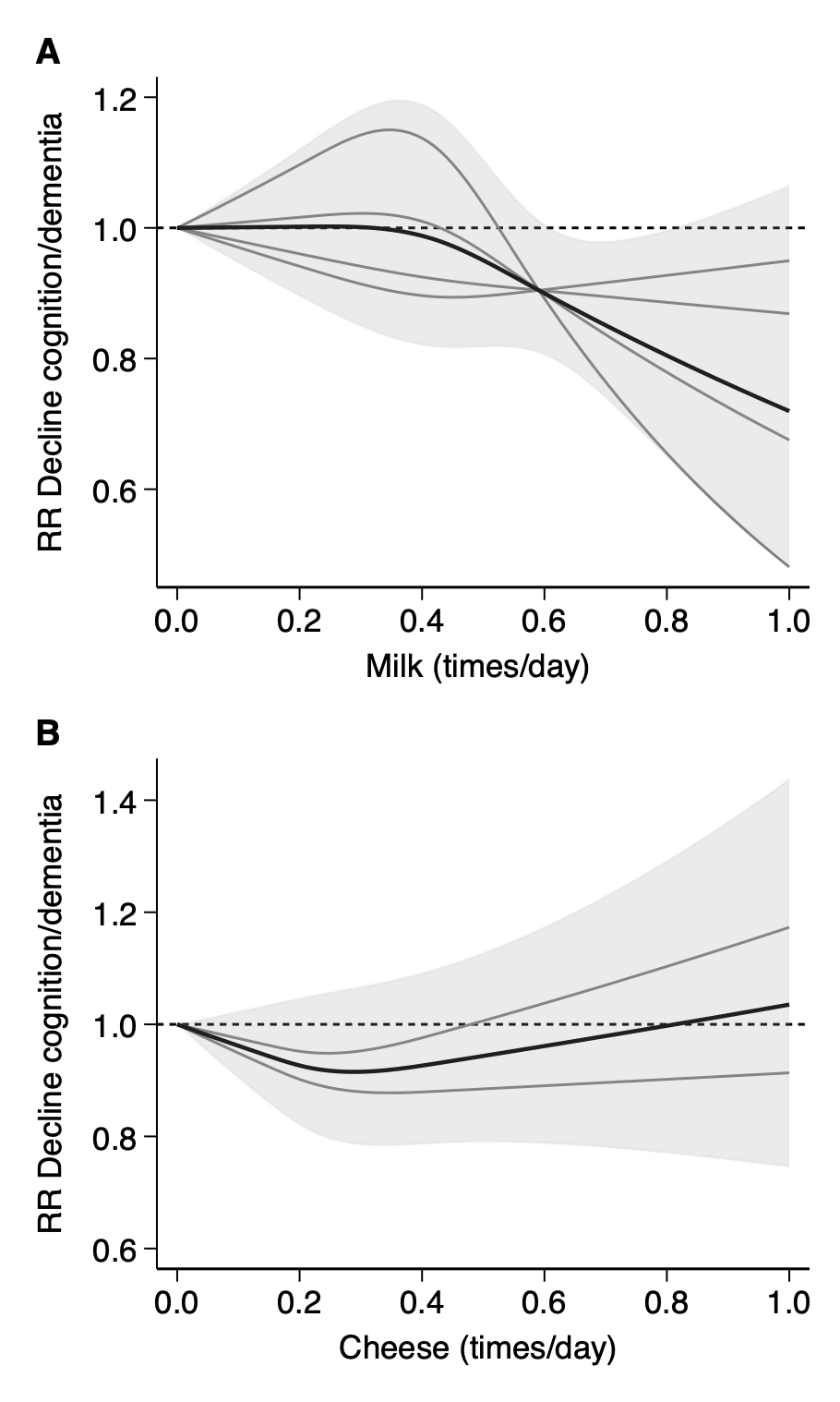

Supplement: Multimedia component1 [file mmc1.docx]
